# Rotational thromboelastometry and conventional coagulation tests in patients undergoing major cardiac or aortic surgery: a retrospective single-center cohort study

**DOI:** 10.1007/s11239-021-02519-y

**Published:** 2021-07-07

**Authors:** Cornelius Keyl, Albina Bashota, Friedhelm Beyersdorf, Dietmar Trenk

**Affiliations:** 1 Department of Anesthesiology, University Heart Center Freiburg - Bad Krozingen, Medical Faculty of the Albert-Ludwigs-University Freiburg, Suedring 15, 79189 Bad Krozingen, Germany; 2 Department of Cardiovascular Surgery, University Heart Center Freiburg - Bad Krozingen, Medical Faculty of the Albert-Ludwigs-University, Freiburg, Germany; 3 Department of Clinical Pharmacology, University Heart Center Freiburg - Bad Krozingen, Medical Faculty of the Albert-Ludwigs-University, Freiburg, Germany

**Keywords:** Blood coagulation tests, Thromboelastography, Bleeding, Intraoperative complications, Cardiac surgical procedures

## Abstract

Algorithms for treatment of diffuse bleeding in cardiac surgery are based on intervention thresholds of coagulation tests, such as rotational thromboelastometry (ROTEM) or conventional laboratory tests. The relationship between these two approaches is unclear in patients with increased risk of coagulation abnormalities. We retrospectively analyzed the data of 248 patients undergoing major cardiac and/or aortic surgery. ROTEM and conventional laboratory tests were performed simultaneously after termination of cardiopulmonary bypass and protamine administration to investigate the extrinsic and intrinsic system, and to determine deficiencies in platelets and fibrinogen. We evaluated the association between ROTEM and conventional tests by linear regression analysis and compared the frequency of exceeding established thresholds for clinical intervention. Significant linear associations between ROTEM 10 min after the start of coagulation, and plasma fibrinogen concentration or platelet count (FIBTEM A10, R^2^ = 0.67, p < 0.001; EXTEM A10, R^2^ = 0.47, p < 0.001) were obtained. However, the 95% prediction intervals exceeded clinically useful ranges (92–233 mg/dL fibrinogen at the intervention threshold of FIBTEM A10 = 10 mm; 14 × 10^3^–122 × 10^3^/µL platelets at the intervention threshold of EXTEM A10 = 40 mm). The association between EXTEM and INR (R^2^ = 0.23), and INTEM and aPTT (R^2^ = 0.095) was poor. The frequency of exceeding intervention thresholds and, consequently, of triggering treatment, varied markedly between ROTEM and conventional tests (p < 0.001 for all comparisons). The predictability of conventional coagulation test results by ROTEM is limited, thus hampering the interchangeability of methods in clinical practice.

**Supplementary Information:**

The online version contains supplementary material available at 10.1007/s11239-021-02519-y.

## Highlights

- Rotational thromboelastometry (ROTEM) and conventional coagulation tests are considered as interchangeable to trigger therapeutic interventions in patients with increased risk of postoperative coagulation abnormalities
- We analyzed the relationship between methods in patients undergoing major cardiac and/or aortic surgery after termination of extracorporeal circulation and protamine administration
- We found a limited predictability of conventional coagulation tests by ROTEM, the 95% prediciton intervals exceeded clinically useful ranges
- The frequency of triggering therapeutic interventions varied significantly between methods
- The evaluation of the therapeutic benefit of ROTEM or conventional tests for decision making is clinically important and should be subject of future research

## Introduction

Rotational thromboelastometry (ROTEM) has been established as a point-of-care method to gain information on the coagulation profile in patients who are at high risk of diffuse bleeding in the intra- and postoperative period. Several algorithms for treatment of coagulation disorders, based on intervention thresholds of conventional coagulation tests or ROTEM, have been published in the last years [1–6]. A number of published investigations suggest a close interrelationship, expressed as statistically significant correlation or as linear regression model, between ROTEM and conventional coagulation parameters, such as plasma fibrinogen concentration, platelet count, international normalized ratio (INR) and activated partial thromboplastin time (aPTT) [7–13]. However, studies evaluating the association between ROTEM and conventional coagulation tests in patients undergoing major cardiac or aortic surgery with high risk of postoperative bleeding, as well as the impact of the coagulation assays on clinical decision-making, are lacking. Therefore, we retrospectively analyzed the data of patients who had undergone major cardiac and/or aortic surgery and who were predisposed to acquired coagulation disorders and bleeding complications. ROTEM and conventional coagulation tests were performed simultaneously after termination of extracorporeal circulation and heparin antagonization by protamine in all patients. Our study tested the hypothesis that there is a significant correlation between the results of ROTEM and conventional coagulation tests, and that plasma fibrinogen concentration, platelet count, INR, and aPTT can be predicted by ROTEM analyses. Furthermore, we tested the hypothesis, that the frequency of exceeding intervention thresholds, which serves as a trigger for therapeutic decisions in patients with ongoing diffuse bleeding, is comparable between ROTEM and conventional coagulation tests.

## Methods

The study was performed as a retrospective single-center cohort study. We analyzed all data from patients 18 years of age or older undergoing major cardiac and/or aortic surgery (redo cardiac surgery, multiple valve surgery, surgery of native or prosthetic valve endocarditis, surgery of the aortic valve with simultaneous replacement of the ascending aorta, surgery of the aortic arch, and replacement of the thoracoabdominal aorta) between January 2015 and February 2019. The study protocol was approved by the local ethics committee who waived the need for obtaining informed consent of patients for this retrospective analysis (application ID 110/19, approved on July 2, 2019).

Data were retrieved from the electronic hospital information system (Patidok, Professional Clinical Software GmbH, Klagenfurt, Austria), and from the electronic patient database (Metavision, IMDsoft, Tel Aviv, Israel).

Routine clinical laboratory assessments were scheduled in all patients before surgery. Blood gas analyses and determination of electrolytes, hemoglobin, hematocrit and lactate were performed on a regular basis in all patients. ROTEM and conventional coagulation tests were performed simultaneously after termination of extracorporeal circulation and administration of protamine.

All coagulation assays (ROTEM and conventional coagulation tests) were performed by experienced laboratory staff in the clinical laboratory of our hospital. Blood samples were delivered by a pneumatic tube system and the results were communicated via the hospital intranet. Results of ROTEM were visualized in real time using the software “ROTEM live”.

The conventional coagulation tests comprised the determination of platelet count by an automated flow cytometry assay (Sysmex XN 1000, Sysmex Norderstedt, Germany), fibrinogen using the Clauss method, prothrombin time, INR, and aPTT by routine laboratory assays (STA R Max, Stago, Düsseldorf, Germany).

ROTEM was performed using ROTEM delta (Werfen, München, Germany). The ROTEM tests comprised EXTEM (a tissue-activated test to analyze the clot formation via the extrinsic pathway), INTEM (a contact-activated test to analyze the clot formation via the intrinsic pathway) and FIBTEM (EXTEM plus the platelet inhibitor Cytochalasin D for analyzing the fibrinogen contribution to the clot). In addition, a contact-activated test with heparinase was used to exclude persisting heparin effects (HEPTEM) in all patients. Parameters used for analysis of coagulation were the time to the start of coagulation (CT) and the amplitude 10 min after the start of coagulation (A10).

The intervention thresholds for triggering therapeutic interventions in bleeding patients followed recommendations and algorithms published previously: extrinsic system, EXTEM CT > 80 s, INR > 1.4; intrinsic system, INTEM CT > 240 s, aPTT > 50 s; fibrinogen, FIBTEM A10 < 10 mm, plasma fibrinogen concentration < 200 md/dL; platelets, EXTEM A10 < 40 mm, platelet count < 100 × 10^3^/µL [1, 4, 14–16].

Fluid management comprised the administration of a balanced crystalloid solution, while artificial colloidal solutions were not used at that time. All patients received tranexamic acid following the dosage regimen published by Nutall et al. [17]. Patients experiencing increased bleeding before termination of extracorporeal circulation were initially transfused according to the clinical decision of the anesthesiologist. After determination of ROTEM and conventional coagulation tests, treatment with blood products (platelets, plasma) and coagulation factors (fibrinogen concentrate, prothrombin complex concentrate) was performed in patients with diffuse bleeding with respect to the clinical picture and the intervention thresholds of tests following the decision of the anesthesiologist and the surgical team.

### Statistical analysis

Statistical analysis was performed using the software packages SPSS Statistics version 27 (IBM, Armonk, New York, USA) and Sigma Plot 13.0 (Systat Software, San Jose, CA, USA). A matrix plot visualizing missing data was created using the R software environment version 3.6.3 and the package “VIM”.

Normal distribution of data was evaluated by visual assessment of the histograms and the probability plots (Q–Q plots). Normally distributed data are reported as mean ± standard deviation, otherwise as median with 25th and 75th percentiles. Categorical variables were compared using Fisher’s exact test for two-by-two tables. Tests were performed as two-sided tests with α = 0.05. The relationship between ROTEM and conventional coagulation tests was analyzed using linear regression analysis with the results of ROTEM as the predictor variable and conventional coagulation tests as the outcome variable. The normal distribution of residuals was evaluated using histograms and probability plots. Residual statistics were performed to detect outliers and influential cases. Cases with studentized residuals > 3 or a critical Mahalanobis distance at p < 0.001 were examined in closer detail. Cases were excluded from analysis, if Cook’s distance was greater than 1, and if they were obviously not plausible clinically. The lack of autocorrelation was tested using the Durbin-Watson test. The data were tested for heteroscedasticity by plotting studentized residuals against the predicted values and by the modified Preusch-Pagan test. In the case of heteroscedasticity, the 95% confidence interval for the regression coefficients was calculated using a bootstrapping process (corrected and accelerated confidence interval based on 1000 bootstrap samples).

The sample size was determined using the software G*Power (Department of Experimental Psychology, Heinrich-Heine-University, Düsseldorf, Germany). The minimally required sample size was calculated to detect a deviation of a single linear regression coefficient from zero, given f^2^ = 0.05, which corresponds to a small to medium effect size, with α = 0.05 and β = 0.1. According to this assumption, we calculated a minimal sample size of 202 patients.

## Results

The study cohort comprised 248 patients in total. The clinical characteristics of the patients are summarized in the Supplementary Table. A matrix plot of the available and missing data of ROTEM and conventional coagulation tests is provided in the Supplementary Figure.

**Fig. 1:**
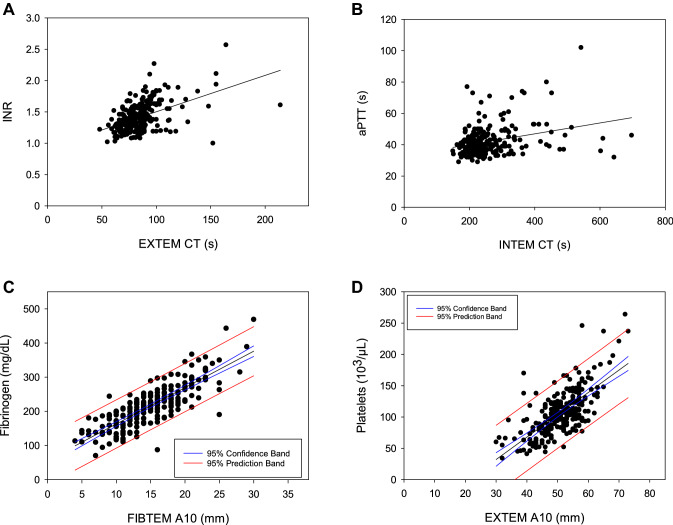
Relationship between EXTEM CT and INR (**A**), INTEM CT und aPTT (**B**), FIBTEM A10 and plasma fibrinogen concentration (**C**), and EXTEM A10 and platelet count (**D**). The 95% confidence intervals and 95% prediction intervals of linear regression are indicated in graph **C** and **D**. Models A and B show heteroscedasticity, standard errors were estimated using bootstrapping. See Table 1 for details of regression analysis

The relationship between ROTEM and conventional coagulation tests is demonstrated in Fig. 1, the results of linear regression analysis are provided in Table 1.

**Table 1:** Details of linear regression analysis

Dependent variable	Independent variable	R^2^	p-value	Durbin-Watson	Unstandardized coefficient (95% CI)	Standardized coefficient
INR	EXTEM CT	0.23	< 0.001	1.74	0.006 (0.003; 0.009)	0.48
aPTT	INTEM CT	0.095	< 0.001	1.76	0.035 (0.013; 0.066)	0.309
Plasma fibrinogen concentration	FIBTEM A10	0.67	< 0.001	1.40	10.66 (9.69; 11.63)	0.82
Platelet count	EXTEM A10	0.47	< 0.001	2.21	3.57 (3.09; 4.06)	0.69

### Relationship between EXTEM CT and INR

Two patients were excluded due to missing values of INR, the results of 3 patients were identified as outliers and excluded from linear regression analysis.

Linear regression analysis revealed a significant relationship between EXTEM CT and INR, but the coefficient of determination was poor (Fig. 1A; Table 1). As the assumption of homoscedasticity was violated, the 95% confidence interval was calculated using bootstrapping.

### Relationship between INTEM CT and aPTT

Four patients were excluded due to missing values (INTEM CT missing in two patients, aPTT missing in two patients), 13 patients were identified as outliers, mainly due to the finding that aPTT had reached the upper limit of the assay (160 s) in 9 of them. Linear regression analysis did not detect a clinically relevant ability of INTEM CT to predict aPTT (Fig. 1B; Table 1). As the assumption of homoscedasticity was violated, the 95% confidence interval was calculated using bootstrapping.

### Relationship between FIBTEM A10 and plasma fibrinogen concentration

Seventeen patients were excluded due to missing values (fibrinogen concentration missing in 15 patients, FIBTEM A10 missing in two patients), one patient was identified as an outlier and excluded from regression analysis. The amplitude of FIBTEM A10 was significantly related to plasma fibrinogen concentration (Fig. 1C; Table 1). The intervention threshold in FIBTEM A10 (= 10 mm) corresponded to a plasma fibrinogen level of 163 mg/dL fibrinogen with a 95% prediction interval of 92 to 233 mg/dL fibrinogen.

### Relationship between EXTEM A10 and platelet count

Eleven patients were excluded due to missing values (platelet count missing in 10 patients, EXTEM A10 missing in one patient), one patient was identified as an outlier and was excluded from linear regression analysis. Linear regression analysis revealed a significant relationship between EXTEM A10 and platelet count, but with a low coefficient of determination (Fig. 1D; Table 1). The intervention threshold of EXTEM A10 (= 40 mm) was related to a platelet count of 68 × 10^3^/µL with a 95% prediction interval of 14 × 10^3^ to 122 × 10^3^/µL.

### Exceedance of intervention thresholds

The intervention thresholds of tests and the results of cross-tabulation analysis, comparing the frequency of data exceeding established intervention thresholds, are presented in Table 2. The frequency of exceeding the intervention threshold was significantly higher in ROTEM—tests evaluating the extrinsic and the intrinsic pathway compared to conventional coagulation assays. Regarding the evaluation of the extrinsic pathway of coagulation, 75 patients had pathological results in both, EXTEM CT and INR, whereas 61 patients (136 minus 75) exceeded the intervention threshold in EXTEM CT solely and 30 patients (105 minus 75) in INR solely. Regarding the assays evaluating the intrinsic pathway of coagulation, 30 patients had pathological results in both, INTEM CT and aPTT, whereas 81 patients exceeded the intervention threshold in INTEM CT solely and 8 patients in aPTT solely.

**Table 2:** Frequency of exceeding intervention thresholds in coagulation tests

		Intervention threshold	Patients exceeding intervention threshold, n (%)	p	Patients exceeding intervention thresholds in both tests, n (%)
Extrinsic system (n = 246)	EXTEM CT	80 s	136 (55.3)	< 0.001	75 (30.5)
	INR	1,4	105 (42.7)		
Intrinsic system (n = 244)	INTEM CT	240 s	111 (45.5)	< 0.001	30 (12.3)
	aPTT	50 s	38 (15.6)		
Fibrinogen (n = 231)	FIBTEM A10	10 mm	26 (11.3)	< 0.001	25 (10.8)
	Plasma fibrinogen concentration	200 mg/dL	92 (39.8)		
Platelets (n = 237)	EXTEM A10	40 mm	16 (6.8)	< 0.001	14 (5.9)
	Platelet count	100 × 10^3^/µL	107 (45.1)		

The frequency of exceeding the intervention threshold was significantly higher in FIBTEM A10 compared to the plasma fibrinogen concentration. Twenty-five patients exceeded the intervention threshold in both, FIBTEM A10 and plasma fibrinogen concentration, whereas a single patient exceeded the threshold in FIBTEM A10 solely, and 67 patients in fibrinogen concentration solely. Using 163 mg/dL instead of 200 mg/dL as the intervention threshold of plasma fibrinogen concentration, as predicted by the linear regression analysis, 45 patients (19.5%), instead of 92 patients (39.8%), exceeded this threshold. Eighteen patients were below the intervention threshold in both, FIBTEM A10 and plasma fibrinogen concentration, whereas 8 patient exceeded the threshold in FIBTEM A10 solely and 27 patients in plasma fibrinogen concentration solely. Recalculating Fisher’s exact test for two-by-two tables, a significant difference in triggering therapeutic interventions between FIBTEM A10 and plasma fibrinogen concentration was found (p < 0.001).

The frequency of exceeding the intervention threshold was significantly higher in the platelet count compared to EXTEM A10. Fourteen patients exceeded the intervention threshold in both, EXTEM A10 and platelet count, whereas 2 patients exceeded the threshold in EXTEM A10 solely, and 93 patients in platelet count solely. When using 68 × 10^3^ platelets/µL instead of 100 × 10^3^ platelets/µL as the intervention threshold, as determined by linear regression analysis, 31 patients (13.1%), instead of 107 patients (45.1%), fell below this threshold. Eleven patients exceeded the threshold in both, EXTEM A10 and platelet count, whereas 5 patients exceeded the threshold in EXTEM A10 solely, and 20 patients in platelet count solely. Recalculating Fisher’s exact test for two-by-two tables, a significant difference in triggering therapeutic interventions between EXTEM A10 and platelet count was found (p < 0.001).

### The ratio HEPTEM CT/INTEM CT

A HEPTEM CT / INTEM CT ratio < 0.66 has been postulated to indicate a remaining heparin effect, i.e., an incomplete antagonization of heparin by protamine [18]. Fourteen patients had a HEPTEM CT / INTEM CT ratio < 0.66 and received additional protamine. Overall, HEPTEM CT was not significantly shorter than INTEM CT (HEPTEM CT/INTEM CT = 1.04 [0.94; 1.18]) in our patients, thus excluding a systematical bias due to a persisting heparin effect.

## Discussion

The current study focused on patients undergoing major cardiac and/or aortic surgery with high risk of postoperative bleeding complications and coagulopathy. ROTEM and conventional coagulation analyses were determined simultaneously at a well-defined time point (after termination of extracorporeal circulation and antagonization of heparin by protamine), which is important for clinical decision-making. Our data demonstrate a limited interrelationship between ROTEM and conventional coagulation tests, which may cause substantial differences in therapeutic decisions depending on the methodology used.

It is well known that results of ROTEM and conventional tests are not directly comparable, as ROTEM measures the viscoelastic properties of whole blood under static conditions, while there is no contribution of platelet function or cellular elements in conventional coagulation tests due to pre-analytical removal of these components [19]. These methodological differences are, for instance, reflected by the varying congruence between coagulation tests based on the Clauss method for determination of plasma fibrinogen concentration and the clot firmness of ROTEM [13, 20]. The disagreement between these methods increased with supranormal values of plasma fibrinogen and after administration of fibrinogen concentrate [20]. In addition, several studies observed an effect of hematocrit on the amplitude of ROTEM, resulting in a higher correlation between ROTEM and plasma fibrinogen concentration with lower hematocrit [21, 22]. Isovolemic hemodilution has different effect on coagulation tests: prothrombin time increased linearly to the dilution of coagulation factors, wheras aPTT, as well as EXTEM CT and INTEM CT, showed a nonlinear response [23].

Nevertheless, published diagnostic pathways postulate clinically significant relationships between EXTEM CT and INR, INTEM CT and aPTT, FIBTEM A10 and plasma fibrinogen concentration, and EXTEM A10 and platelet count [1, 3, 4, 7–11], and there is an ongoing discussion whether the results of ROTEM and conventional coagulation tests may be regarded as interchangeable [19, 24].

We did not observe a clinically useful relationship between EXTEM CT and INR, and between INTEM CT and aPTT. Although there were statistically significant correlations between the results of ROTEM and the coagulation assays INR and aPTT, which have already been described in previous studies [7, 8, 10], our data demonstrate that a clinically applicable prediction of INR or aPTT by ROTEM is impossible, thus reinforcing comparable conclusions drawn by Haas et al. in a pediatric population [25]. Less than half of our patients with a pathological result of either ROTEM or INR exceeded the intervention thresholds in both tests. As a consequence, therapeutic implications differ substantially, depending on whether ROTEM or INR would be used to trigger treatment. Therefore, our data do not support approaches using ROTEM and INR as interchangeable indicators for the administration of prothrombin complex concentrate [1, 26]. Likewise, ROTEM and aPTT cannot be used as equivalent triggers for therapeutic interventions, as applied in a previous study [5]. Instead, our findings are comparable to the observations of Prakash et al., who reported a lack of interrelationship between INTEM CT and aPTT in patients who were anticoagulated with heparin for extracorporeal membrane oxygenation [27].

We determined a rather wide 95% prediction interval for the forecast of plasma fibrinogen concentration by FIBTEM. In addition, the plasma fibrinogen concentration associated with the intervention threshold of FIBTEM A10 differed markedly from previous assumptions. Previous studies postulated that the intervention threshold of FIBTEM A10 = 10 mm corresponds to a plasma fibrinogen concentration of approximately 200 mg/dL [4, 7], but we observed a substantially lower value (163 mg/dL), comparable to the findings demonstrated by Mace et al. [11]. Using 163 mg/dL instead of 200 mg/dL as the intervention threshold of plasma fibrinogen concentration, 45 patients (19.5%) instead of 92 patients (39.8%) were below this threshold. Nevertheless, there was still a significant difference in triggering therapeutic interventions between ROTEM and plasma fibrinogen concentration. As a consequence, the clinical indication for fibrinogen substitution would have been inconsistent in most of the patients suspicious of fibrinogen deficiency, which again demonstrates the lack of interchangeability between the methods.

Even more pronounced inconsistencies were determined when analyzing the relationship between ROTEM and platelet count. As stated previously, viscoelastic test results are influenced by platelet function, which is regularly impaired after cardiac surgery with extracorporeal circulation [28, 29]. The impact of platelet count and platelet function on ROTEM amplitude has been discussed controversially: whereas some authors proposed the predictability of platelet count by ROTEM, others questioned this approach [30, 31].

The intervention threshold of platelet count in patients with diffuse bleeding varies markedly between publications, ranging from 50 × 10^3^ to 100 × 10^3^ platelets/µL [1, 3, 15, 32, 33]. In accordance with previously published transfusion protocols, we defined EXTEM A10 < 40 mm and a platelet count < 100 × 10^3^ platelets/µL as intervention thresholds for triggering the transfusion of platelets in patients with diffuse bleeding [4, 14, 15, 34]. In contrast, Kuiper et al. and Weber et al. proposed an association between EXTEM A10 = 40 mm and a platelet count of 80 × 10^3^ platelets/µL [1, 32], which seems to be comparable with the observation of Ogawa et al. [7]. However, the platelet count predicted by EXTEM A10 = 40 mm was much lower in our patients, namely 68 × 10^3^ platelets/µL. Furthermore, the large scattering of values, associated with a wide prediction interval, contradicted a clinically useful prediction of platelet count by ROTEM, as proposed previously [30], in our patients. As a consequence, the therapeutic decision to transfuse platelets would vary markedly in our patient cohort, depending on the test method used for decision support.

Up to now, it is unknown which laboratory methods, viscoelastic tests or conventional coagulation tests, or a combination of these assays, are preferable to monitor perioperative coagulation disorders [19, 24, 35, 36]. An essential factor for initiating adequate treatment is the time lag between drawing the blood sample and receiving the test results. The results of ROTEM, visualized in real time via the hospital intranet, can be acknowledged after 10 to 15 min, whereas results of conventional coagulation analyses are frequently delayed in time (20–30 min and longer), depending on local logistic factors. It may be hypothesized that the rapid availability of test results might be more important than the test assay used, as recently emphasized by Schmidt et al. [19].

## Limitations

This retrospective analysis was not designed to evaluate the question as to whether viscoelastic tests, such as ROTEM, conventional coagulation tests, or a combination of these assays, are better suited for predicting postoperative bleeding, or for guiding transfusion therapy and replacement of coagulation factors. The replacement of blood products and coagulation factors in extensive cardiac and vascular surgery is a dynamic process, which, at best, is aimed to prevent the manifestation of severe coagulation and bleedings disorders and, at the same time, to avoid overtreatment and unnecessary use of resources. No standardized procedure depending solely on the results of ROTEM or the results of conventional coagulation analysis was mandatory in our clinical practice, and it was the responsibility of the anesthesiologist and the surgical team to take therapeutic decisions with respect to the clinical picture, as well as the test results.

## Conclusion

In conclusion, our data demonstrate a limited predictability of conventional coagulation tests by ROTEM in clinical practice, thus hampering the interchangeability of methods. The frequency of triggering therapeutic interventions varies significantly between ROTEM and conventional coagulation assays. Controlled studies demonstrating the superiority of either approach in the treatment of bleeding patients are required, but are lacking up to now.

## Supplementary Information

Below is the link to the electronic supplementary material.

Supplementary file1 (EPS 149 kb). Supplementary Figure Matrix plot, visualizing the results of coagulation analyses. Available data are coded in acontinuous grey scale, missing values are indicated in red

Supplementary file2 (DOCX 14 kb)

## Data Availability

The data that support the findings of this study are available from the corresponding author upon reasonable request.
