# Supplementary material for: Rotational thromboelastometry and conventional coagulation tests in patients undergoing major cardiac or aortic surgery: a retrospective single-center cohort study
Source: J Thromb Thrombolysis. 2021 Jul 7;53(1):149–57. doi: 10.1007/s11239-021-02519-y (PMC8791921; doi:10.1007/s11239-021-02519-y)
Supplement: Supplementary file 2 — Supplementary file2 (DOCX 14 kb) [file 11239_2021_2519_MOESM2_ESM.docx]

**Supplementary Table**

Characteristics of Patients (n = 248)

| Age, years | 65 ± 13 |
| --- | --- |
| BMI, kg m^-2^ | 26.2 ± 4.4 |
| Male, n (%) | 162 (65.3) |
| **Preoperative characteristics** | |
| Hypertension, n (%) | 76 (71.0) |
| Diabetes, n (%) | 31 (12.5) |
| Coronary artery disease, n (%) | 82 (33.2) |
| Left ventricular function moderately or severely impaired (LVEF < 40%), n (%) | 25 (10.1) |
| Right ventricular function impaired, n (%) | 30 (12.1) |
| Platelet inhibitors, n (%) | 53 (21.4) |
| Direct oral anticoagulant drug, n (%) | 32 (12.9) |
| **Preoperative laboratory parameters** | |
| Hemoglobin, g/dL | 13.6 [11.9; 14.6] |
| Platelets, 10^3^/µL | 195 [156; 239] |
| INR | 1.07 [1.00; 1.18] |
| aPTT, s | 31 [29; 36] |
| Fibrinogen, g/dL | 309 [251; 396] |
| Creatinine clearance, ml/min | 74 [56; 89] |
| Bilirubin, mg/dL | 0.6 [0.4; 0.9] |
| ALT, U/L | 20 [15; 31] |
| **Intraoperative characteristics** | |
| Emergency surgery n (%) | 58 (23.4) |
| Type of surgery | |
| Multiple valve surgery (except reoperation or endocarditis), n (%) | 31 (12.5) |
| Reoperation, n (%) | 14 (5.6) |
| Aortic valve surgery and ascending aortic replacement, n (%) | 47 (19.0) |
| Aortic arch surgery, n (%) | 111 (44.8) |
| Thoracoabdominal aortic replacement, n (%) | 29 (11.7) |
| Endocarditis, n (%) | 16 (6.5) |
| Surgery duration, min | 308 [263; 378] |
| Cardiopulmonary bypass duration, min | 156 [123; 194] |
| Minimum temperature, °C | 32.6 [25.6; 35.5] |
| Vasopressors/inotropes after cardiopulmonary bypass | |
| Dobutamine, n (%) | 134 (54.0) |
| Epinephrine, n (%) | 79 (31,6) |
| Norepinephrine, n (%) | 232 (93.5) |
| Levosimendan, n (%) | 12 (4.8) |
| Vasopressin, n (%) | 31 (12.5) |
| Mechanical assist device, n (%) | 8 (3.2) |

Data are reported as mean ± standard deviation, as median [25th; 75th percentiles], or as number of patients (%). ALT, alanine aminotransferase; aPTT, activated partial thromboplastin time; BMI, body mass index; INR, international normalized ratio; LVEF, left ventricular ejection fraction; NOAC, new oral anticoagulant drug.
